# What is the Oral Health-related Quality of Life following Miniscrew-Assisted Rapid Palatal Expansion (MARPE)? A prospective clinical cohort study

**DOI:** 10.1186/s12903-022-02444-3

**Published:** 2022-09-22

**Authors:** Aldin Kapetanović, René R. M. Noverraz, Stefan Listl, Stefaan J. Bergé, Tong Xi, Jan G. J. H. Schols

**Affiliations:** 1 Department of Dentistry - Orthodontics and Craniofacial Biology, Radboud Institute for Health Sciences, Radboud University Medical Center, PO Box 9101, 6500 HB Nijmegen, The Netherlands; 2 Department of Dentistry - Quality and Safety of Oral Healthcare, Radboud Institute for Health Sciences, Radboud University Medical Center, PO Box 9101, 6500 HB Nijmegen, The Netherlands; 3 Department of Oral and Maxillofacial Surgery, Radboud Institute for Health Sciences, Radboud University Medical Center, PO Box 9101, 6500 HB Nijmegen, The Netherlands

**Keywords:** Miniscrew-Assisted Rapid Palatal Expansion (MARPE), Maxillary expansion, Oral Health-related Quality of Life (OHRQoL), Oral Health Impact Profile (OHIP), Pain assessment, Visual Analogue Scale (VAS)

## Abstract

**Background:**

Miniscrew-Assisted Rapid Palatal Expansion (MARPE) is a non-surgical orthodontic treatment for transverse maxillary deficiency. This study aimed to investigate the Oral Health-related Quality of Life (OHRQoL) and pain perception of patients undergoing MARPE treatment.

**Methods:**

42 consecutive patients (9 men, 33 women) from the age of 16 onwards (mean: 27.4 ± 9.3 years; range 17.1–55.7 years) who received a MARPE treatment were included. OHRQoL was assessed with the short form of the Oral Health Impact Profile (OHIP-14) questionnaire. Patients filled out the questionnaire at baseline (T0) and weekly during the expansion phase (P1) and in the post-expansion phase (P2). Pain intensity was assessed with a Visual Analogue Scale (VAS) questionnaire and filled out daily during expansion, along with a question on the intake of analgesics. The mean weekly and total OHIP-score and OHIP-score per domain were calculated at T0, P1 and P2, as well as mean weekly and total VAS-scores for average pain, maximum pain and analgesics intake during P1. Kruskal–Wallis tests were used to test for differences in OHIP between T0, P1 and P2. The level of significance was set at 0.05.

**Results:**

The mean OHIP-score was 10.86 ± 9.71 at T0 and increased to 17.18 ± 10.43 during P1 (p < 0.001), after which it returned to pre-expansion levels, 9.27 ± 7.92 (p = 0.907) during P2. At the domain level, there was a statistically significant increase in OHIP-score at P1 for functional limitation, physical pain, psychological discomfort and social disability. The mean VAS-score for average pain during expansion was 16.00 ± 19.73 mm. Both OHIP-score (25.00 ± 10.25), average pain (33.72 ± 16.88 mm), maximum pain (44.47 ± 17.99 mm) and analgesics intake (59%) were highest at initiation of the expansion and decreased by the end of expansion.

**Conclusions:**

MARPE is a generally well-tolerated expansion treatment. A temporary decline in OHRQoL and moderate pain are present at the start of expansion, followed by a recovery of OHRQoL and very mild pain during the rest of treatment. Clinicians should be aware of the effects of MARPE on patients’ quality of life and manage the expected discomfort and impediments with adequate communication and patient education.

## Background

Transverse maxillary deficiency is a common orthodontic problem, with a prevalence of ca. 10% in adults [1]. Rapid Palatal Expansion (RPE) is an effective treatment for children and young adolescents, while older adolescents and adults usually need Surgically-Assisted Rapid Palatal Expansion (SARPE) as the opening of an increasingly interdigitated midpalatal suture requires higher forces [2, 3]. In recent years, Miniscrew-Assisted Rapid Palatal Expansion (MARPE) has positioned itself as a non-surgical alternative. It is reported to be an effective treatment for maxillary expansion in patients from the age of 16 onwards (success rate: 92.5%), with limited side effects and a relatively short duration [4].

Beside objective measurements of treatment effect, the Oral Health-related Quality of Life (OHRQoL), a multidimensional concept assessing patient-reported outcome measures of psychosocial impact, orofacial pain, oral functions and appearance, has been recognized as essential to evaluate the impact of new therapies [5, 6]. OHRQoL can be measured with different tools, the most common one being the validated Oral Health Impact Profile (OHIP) questionnaire [7]. Previous systematic reviews have found evidence that malocclusion has an adverse effect on OHRQoL, whereas the completion of orthodontic treatment leads to an improvement [8, 9]. Furthermore, there is evidence that patient perception is a key determinant of treatment success, as pain and discomfort during orthodontic treatment, such as maxillary expansion with (SA)RPE, are found to negatively impact the OHRQoL, reducing patient motivation, treatment compliance and satisfaction with the treatment outcome [10–14].

Considering the interrelationship between treatment success and OHRQoL during expansion treatment, it would be appropriate to assess the quality of life following MARPE when evaluating this relatively novel expansion technique. Clinicians ought to be aware of the physical, psychological and social effects and the intensity of pain and discomfort that patients perceive in order to improve pain management, patient experience and education, and this should be systematically evaluated. Yet, a recent literature search revealed a lack of scientific literature on the topic of OHRQoL and pain perception during maxillary expansion with MARPE.

The primary aim of the present study was to evaluate the OHRQoL outcomes following MARPE treatment. The secondary aim was to evaluate the pain intensity during MARPE.

## Methods

A prospective cohort study was set up in October 2018 and approved by the Radboud University Medical Centre Institutional Review Board (IRB no. 2018-4083). A written informed consent was obtained from all participants.

**Fig. 1:**
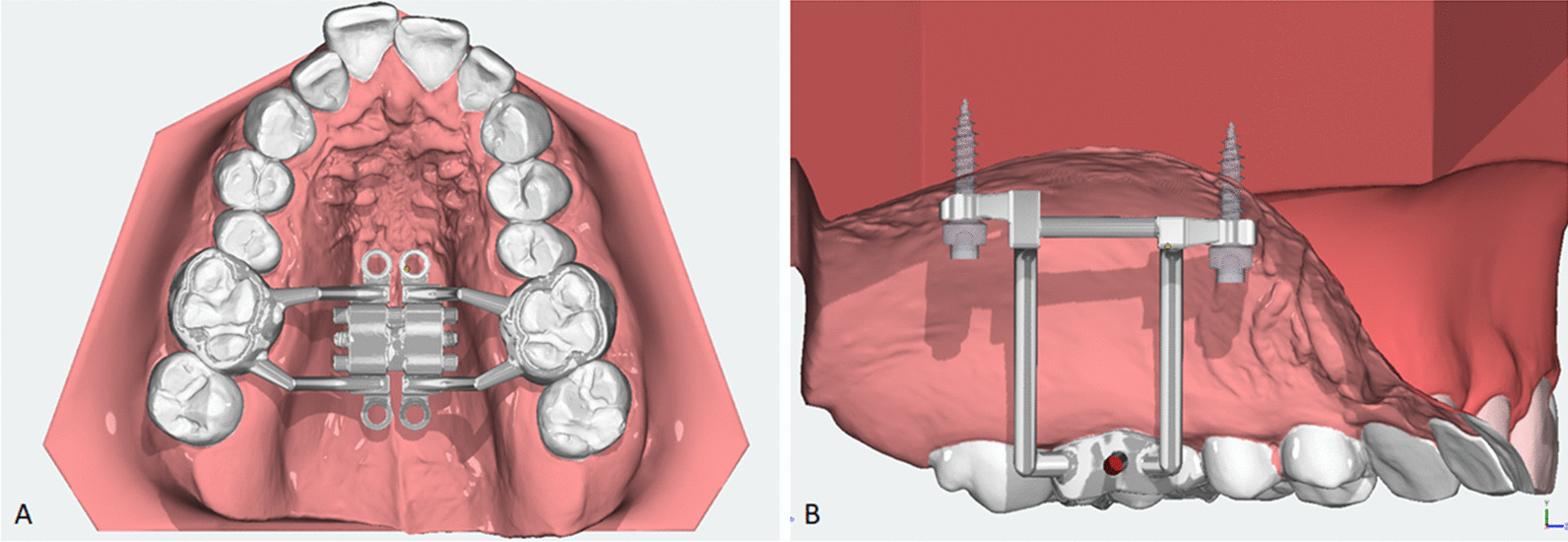
The Dutch Maxillary Expansion Device (D-MED) on intra-oral scan. Occlusal view of the digital D-MED design (**A**) and miniscrew positioning with the D-MED (**B**)

**Fig. 2:**
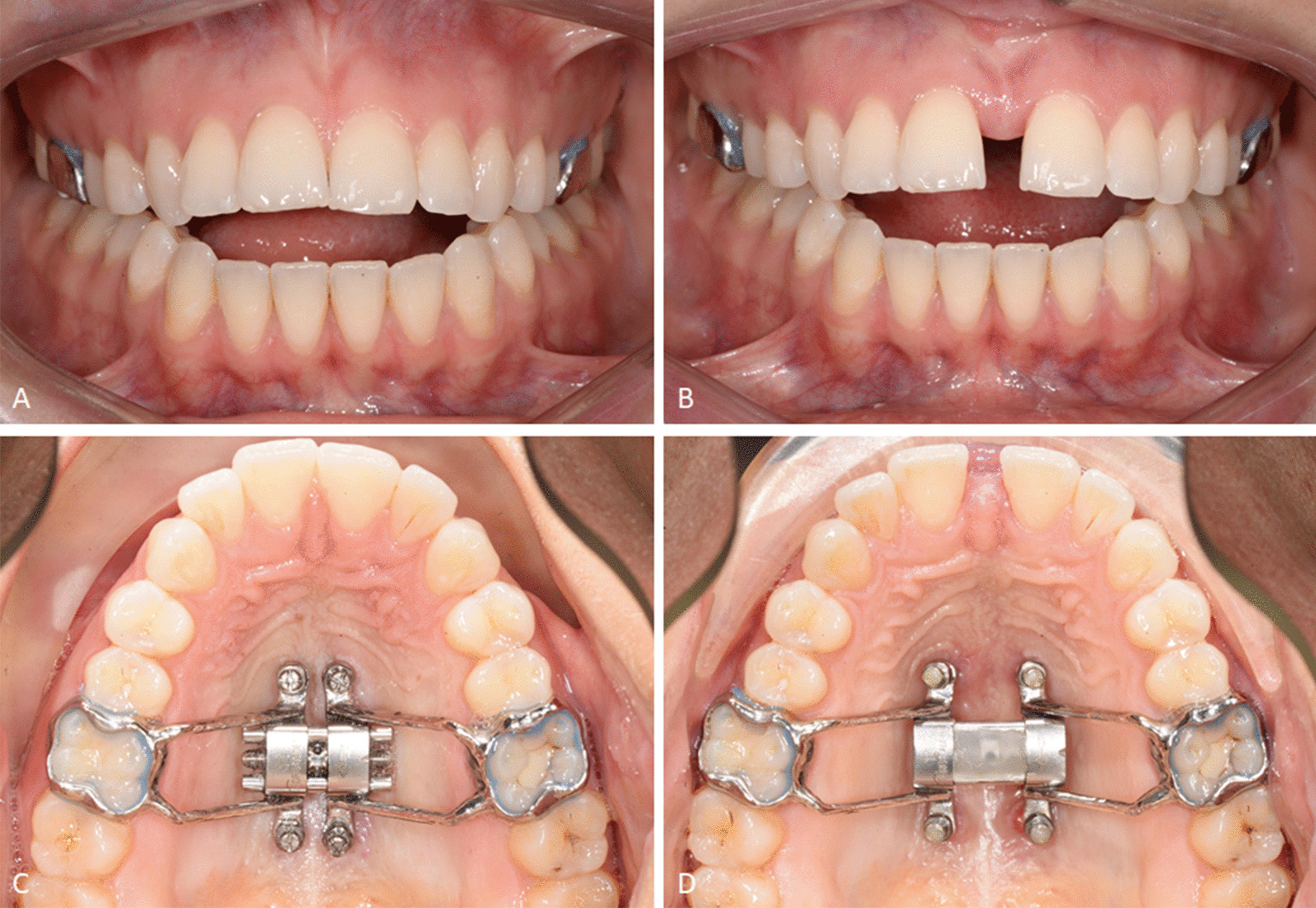
Intra-oral photographs of a patient pre- and post-expansion with the D-MED. Frontal view before (**A**) and after (**B**) expansion. Maxillary occlusal view before (**C**) and after (**D**) expansion

The patients that qualified under the eligibility criteria of the study received an expansion treatment with MARPE. The inclusion criteria were consecutive patients from the age of 16 years and older, without upper age limit, who presented with transverse maxillary discrepancy and needed expansion. The transverse maxillary discrepancy was evaluated intra-orally during the oral exam and could present as a unilateral, bilateral or anticipated crossbite, or maxillary constriction without crossbite. The exclusion criteria were patients with a history of maxillofacial surgery, cleft lip and palate, craniofacial anomalies or syndromes, congenital tooth anomalies, absent first or second molars or extensive prosthetic restorations in the molar region. The patients were treated with the Dutch Maxillary Expansion Device (D-MED) (Radboudumc, Nijmegen, The Netherlands & Orthoproof, Nieuwegein, The Netherlands), an individualised, 3D-designed and fabricated tooth-bone borne MARPE appliance, which has demonstrated a very high success rate and amount of skeletal expansion. The D-MED consisted of a stainless-steel structure, including two bands around the upper first molars and four rigid connectors with screw holes for the miniscrews that anchor the device to the palate, and an expansion screw soldered to the base of the structure (see: Fig. 1). It was positioned parallel to the palate at the level of the upper first molars and was activated according to protocol, by one turn of the expansion screw per day, until the required amount of expansion was achieved (see: Fig. 2) [15].

The participants were asked to fill out two online questionnaires throughout the treatment, with the aim of assessing 1) OHRQoL following MARPE, together with its functional, physical, psychological and social dimensions and 2) pain intensity and intake of analgesics. Castor Electronic Data Capture (EDC) System for clinical research (Castor*,* Amsterdam, The Netherlands. Available at https://www.castoredc.com) was used to design, send and collect the questionnaires. This cloud-based clinical data management platform allowed processing and storage of data in a secure and anonymised way, compliant to GDPR. All amendments of data were logged in Castor EDC.

### Oral Health-related Quality of Life assessment

The OHRQoL was assessed by the short form of the OHIP-questionnaire. The short form consists of 14 questions, versus 49 in the long form, and is easier and more user-friendly to fill out, while demonstrating equivalent validity compared to the long form [16]. The Dutch translation of OHIP-14 was used in this study, OHIP-NL14, which has been translated and validated by van der Meulen et al. (2011), showing excellent reliability and validity. [17]. The OHIP evaluated seven domains: functional limitation, physical pain, psychological discomfort, physical disability, psychological disability, social disability, and handicap. (see: Table 1) The questions measured incidence of oral health-related problems during the past week and were rated on a 5-point Likert scale, with possible scores 0: “Never”, 1: “Hardly ever”, 2: “Occasionally”, 3: “Fairly often” and 4: “Very often”. The sum resulted in a total score between 0 and 54, with a higher score indicating a worse OHRQoL [16]. The patients received a digital OHIP-NL14 questionnaire at set times:

- At baseline (T0): on day 1 of treatment, right before placing of the MARPE
- During the expansion phase (P1): weekly, on day 8, 15, 22… from the start until the completion of expansion. The duration of this phase varied due to differences in the required amount of maxillary expansion and the speed of the midpalatal suture opening among patients.
- During the retention phase (P2): weekly, on day 1, 8, 15… for 12 weeks: from the completion of expansion until the placing of the orthodontic upper fixed appliance

**Table 1:** OHIP-14: the 7 domains and 14 corresponding questions

OHIP domain	OHIP questions “In the past week, have you… because of problems with your teeth, mouth or dentures?”
Domain 1. Functional limitation	1. Had trouble pronouncing any words
	2. Felt that your sense of taste has worsened
Domain 2. Physical pain	3. Had painful aching in your mouth
	4. Found it uncomfortable to eat any foods
Domain 3. Psychological discomfort	5. Felt self-conscious
	6. Felt tense
Domain 4. Physical disability	7. Has your diet been unsatisfactory
	8. Had to interrupt meals
Domain 5. Psychological disability	9. Found it difficult to relax
	10. Been a bit embarrassed
Domain 6. Social disability	11. Been a bit irritable
	12. Had difficulty doing your usual jobs
Domain 7. Handicap	13. Felt that life in general was less satisfying
	14. Been totally unable to function

For the data analysis, the following primary outcome measures were included:

- Mean total OHIP-score and score per domain at T0
- Mean total and weekly OHIP-score and score per domain during P1
- Mean total OHIP-score during P2

### Pain intensity assessment

**Fig. 3:**
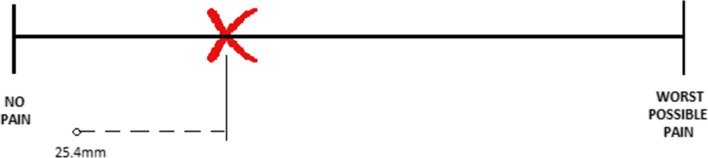
The Visual Analogue Scale (VAS) as a pain assessment tool measuring pain intensity

Physical pain was assessed as a domain of the OHIP-questionnaire, measuring the incidence of painful aching and discomfort while eating. However, this assessment did not provide treatment-relevant information about the intensity of pain experienced by patients. Therefore, a separate pain assessment was conducted in the form of a single-dimensional pain scale: the Visual Analogue Scale (VAS), a 100-mm line on which patients would mark their pain intensity, with “no pain” at the left end and “the worst imaginable pain” at the right end, corresponding to a pain score between 0 and 100 (see: Fig. 3). It is a validated tool for measuring pain intensity in situations of acute pain when the aetiology is clear, as well as for the evaluation of potential fluctuations and clinically relevant intraindividual changes for pain intensity [18, 19].

The patients received a pain questionnaire and were asked to fill it out at the end of each day (on day 1, 2, 3…) from the start until the completion of the expansion with MARPE (P1), with the number of days varying due to differences in the required amount of maxillary expansion and the speed of the midpalatal suture opening among patients. The questionnaire included three questions:

- “How intense was the average pain you had today?”
- “How intense was the maximum pain you had today?”
- “Did you take any analgesics today?”

The patients were asked to mark their perceived pain intensity on the VAS, while for the scoring of analgesics intake, a binary choice was used: 0, no analgesics taken, or 1, analgesics taken. To correct for the variation in the length of the expansion phase among patients, the duration of expansion was divided into fifths and the mean VAS-score for average pain, maximum pain and the score for analgesics intake was calculated for each fifth, or 20th percentile, of the expansion phase. For the data analysis, the following secondary outcome measures were included, for the expansion per week and for each fifth of expansion:

- Mean VAS-score for intensity of the average pain during P1
- Mean VAS-score for intensity of the maximum pain during P1
- Mean score for analgesics intake by patients during P1.

### Data analysis and statistics

This study was reported conform the STROBE guidelines for reporting of observational studies. All data were collected and anonymised in Castor EDC, by assigning a serial number to each patient. The code was stored in a password-protected environment and was only accessible to the main researcher. In case a questionnaire was not completed, participants would receive a weekly reminder to fill it out. In case there would be no reply, it would be coded as missing data. Data were extracted from Castor by one blinded assessor (A.K.) in order to perform the data analysis. The VAS- and OHIP-scores were explored with descriptive statistics. Normality of data was tested with Shapiro–Wilk tests and Kruskal–Wallis tests were used to evaluate differences in OHIP between T0, P1 and P2. The level of significance was set at 0.05. Given that there were no similar studies in the literature, an adequate sample size calculation could not be performed. The study sample included patients undergoing maxillary expansion with MARPE in the context of a study concerning its efficacy. The power analysis was based on the success of MARPE expansion and resulted in a sample size of 33 patients (alpha: 0.05, power: 95%, effect size: 0.66) [15]. All statistical analyses were performed using SPSS® Statistics version 25.0 (IBM Corp., Armonk, NY, USA).

## Results

Forty-five consecutive patients received a MARPE treatment with the Dutch Maxillary Expansion Device. Three patients were excluded: one who chose to terminate the orthodontic treatment after two days and two who needed a second MARPE device to achieve sufficient expansion. Finally, 42 patients (9 men, 33 women) with a mean age of 27.4 ± 9.3 years old (age range 17.1–55.7 years) and a mean expansion duration (P1) of 33.6 ± 11.4 days (range 21–70 days) were included. Forty of them were successful, achieving the required amount of maxillary expansion without the need for a surgical intervention (SARPE). The expansion failed in two patients (2 men; 26.4 and 56.0 years old) and was discontinued, but they were included in the study group because the expansion procedure did not differ from the successful group. From 42 patients, 7 achieved the required amount of maxillary expansion and completed the treatment after 3 weeks, while 35 patients continued expanding. After 4 weeks, 12 more patients completed their expansion and 23 patients continued, while after 5 weeks, another 14 patients ended the treatment, together representing the vast majority of 33 out of 42 or 78.6% of patients, who completed their expansion within 5 weeks. The remaining 9 patients continued expanding until the 6th week, when 2 finished; 7 patients continued until the 7th week, when another 2 finished; 5 patients until the 8th week, when 4 finished; and one last patient expanded until the 10th week in order to achieve the required amount of expansion.

### Oral Health-related Quality of Life

**Fig. 4:**
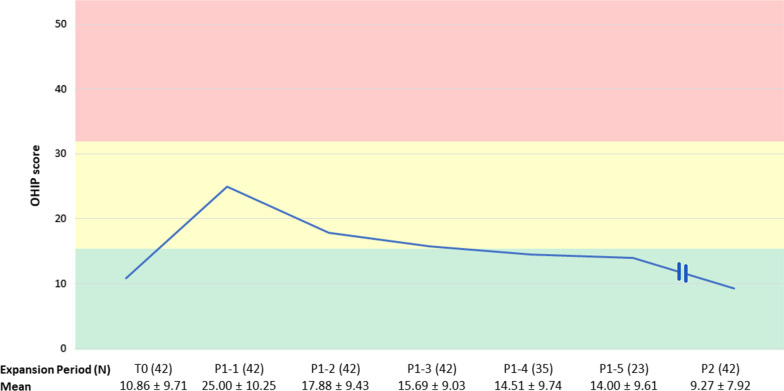
Results of the OHIP-14 questionnaires. Mean OHIP-score ± SD and the sample size (N) are shown for T0, the first five weeks during P1, and for P2. The green bar represents a mild, the yellow bar a moderate and the red bar a severe negative impact on OHRQoL

The response rate for the OHIP-NL14 was 100%. The results for the OHIP-scores at T0, for every week during P1, and for P2 are shown in Table 2. The mean OHIP-score was 10.86 ± 9.71 at T0 and increased significantly to a mean score of 17.18 ± 10.43 during P1 (p < 0.001). During P2, the mean OHIP-score was 9.27 ± 7.92, a significant decrease from P1 (p < 0.001). There was no significant difference in OHIP-score between T0 and P2 (p = 0.907). At the week level, during P1, the mean OHIP-score was highest at the start of expansion, first week: 25.00 ± 10.25 (N = 42), and decreased by the end of expansion. Figure 4 shows the results for T0, the first 5 weeks of P1 (N = 33), and P2.

**Table 2:** Results of the OHIP and pain questionnaires

	T0	P1										P2
		Week 1	Week 2	Week 3	Week 4	Week 5	Week 6	Week 7	Week 8	Week 9	Week 10	
N	42	42	42	42	35	23	9	7	5	1	1	42
Mean OHIP-score	10.86 ± 9.71	25.00 ± 10.25	17.88 ± 9.43	15.69 ± 9.03	14.51 ± 9.74	14.00 ± 9.61	12.67 ± 8.38	14.29 ± 12.76	9.80 ± 6.65	4.00	0.00	9.27 ± 7.92
Mean VAS-score for average pain (mm)		34.38 ± 23.72	17.97 ± 17.31	10.66 ± 13.78	8.83 ± 14.83	8.81 ± 13.48	9.83 ± 17.42	6.18 ± 11.35	2.68 ± 5.08	0.00 ± 0.00	2.86 ± 4.98	
Mean VAS-score for maximum pain (mm)		44.33 ± 27.01	26.43 ± 21.64	16.68 ± 18.79	14.00 ± 19.54	14.37 ± 17.45	14.37 ± 22.38	13.24 ± 18.72	4.87 ± 5.98	0.00 ± 0.00	3.71 ± 5.19	
Mean score for analgesics intake (%)		56 ± 50	20 ± 40	14 ± 35	10 ± 30	10 ± 30	14 ± 35	8 ± 28	0 ± 0	0 ± 0	0 ± 0	

The OHIP-score ± SD at T0, for each week of P1 and for P2 are shown, as well as the sample size (N) per time point. p < 0.001 for mean OHIP change T0–P1 and P1–P2; p = 0.907 for mean OHIP change T0–P2, as calculated with the Kruskal–Wallis test after the Shapiro–Wilk test showed non-normal distribution of data. The VAS-score ± SD (mm) for average pain, maximum pain and the score for analgesics intake ± SD (%) are shown for each week of P1. The data of every 7 consecutive daily pain questionnaires were pooled and mean weekly scores for pain and analgesics intake were calculated. The VAS-score ± SD (mm) for average pain, maximum pain and the score for analgesics intake ± SD (%) are shown for each week of P1

**Fig. 5:**
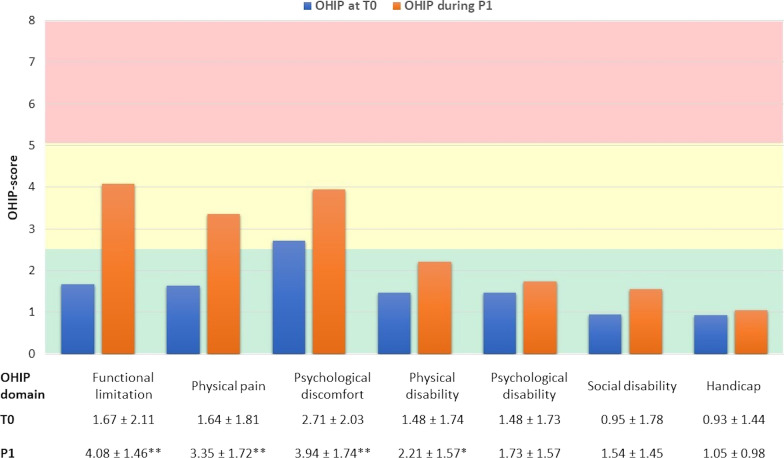
Results of the OHIP-14 questionnaires per domain. Mean OHIP-score ± SD per domain is shown for T0 and P1. Kruskal–Wallis tests were performed to determine the level of significance, with *p < 0.05 and **p < 0.001. The green bar represents a mild, the yellow bar a moderate and the red bar a severe negative impact on OHRQoL

At the OHIP-domain level, there was a statistically significant increase in OHIP-score for 4 domains: functional limitation, physical pain, psychological discomfort and social disability during expansion. The results and a graph for the OHIP-scores per domain are shown in Fig. 5.

### Pain intensity

**Fig. 6:**
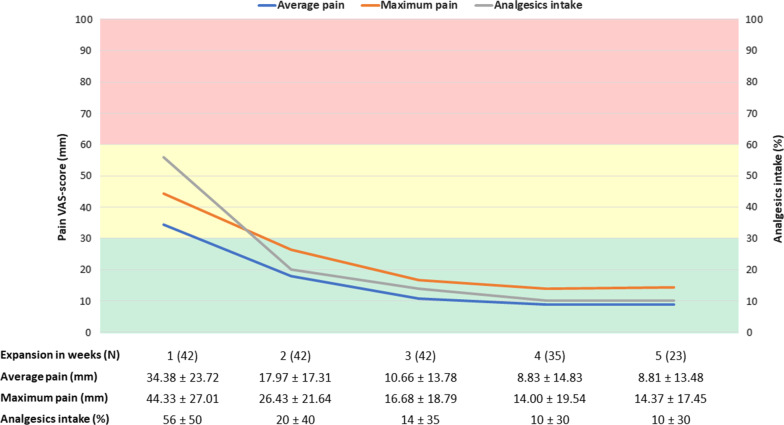
Results of the pain questionnaires. Mean VAS-scores ± SD (mm) and the sample size (N) for the first 5 weeks of P1 for average pain, maximum pain and the score for intake of analgesics (%) are shown. The green bar represents mild, the yellow bar moderate and the red bar severe pain

The response rate for the pain questionnaires was 100% and the mean results per week during P1 are shown in Table 2. The mean VAS-score for average pain intensity during P1 was 16.00 mm ± 19.73 mm, while the mean maximum pain was 23.55 mm ± 24.39 mm and the score for intake of analgesics was 0.22 ± 0.42, or 22%. At the week level, both average pain (34.38 ± 23,72 mm), maximum pain (44.33 ± 27.01 mm) and analgesics intake (0.56 ± 0.50, or 56%) were highest at initiation, week 1 (N = 42), of the expansion and gradually decreased by the end of P1. All patients (N = 42) used analgesics during expansion and the intake of analgesics decreased at a faster pace than the pain intensity. Figure 6 shows the results for the first 5 weeks of the expansion phase (N = 33).

**Fig. 7:**
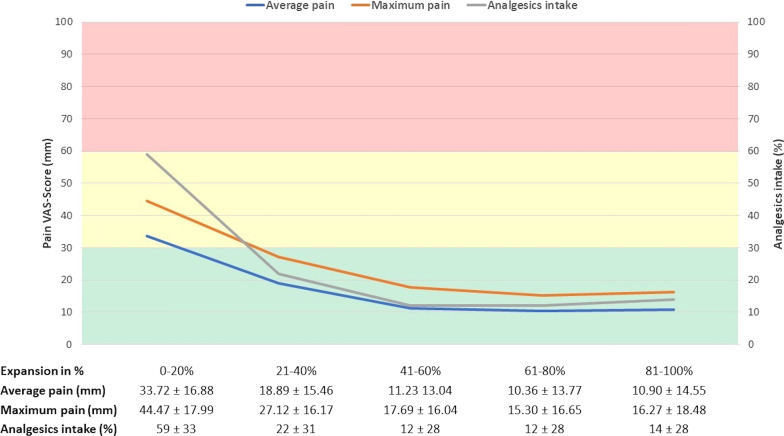
Results of the pain questionnaires per 20th percentile (fifth) of expansion. Mean VAS-scores ± SD (mm) for average pain, maximum pain and the score for intake of analgesics (%) are shown per 20th percentile of expansion duration. The green bar represents mild, the yellow bar moderate and the red bar severe pain

Given that the mean duration of P1 was 33.6 ± 11.4 days, each fifth, or 20th percentile, of the expansion phase corresponded with a duration of 6.72 days. The mean VAS-scores per fifth of the expansion duration for average pain, maximum pain and the score for intake of analgesics are shown in Fig. 7.

## Discussion

### Oral Health-related Quality of Life

The OHIP-(NL)14 is a validated questionnaire designed to measure self-perceptions of patients regarding their oral health and well-being [16, 17]. The response rate in this study was very high owing to the use of frequent reminders. The results showed that patients in need of maxillary expansion had a mildly decreased OHRQoL ahead of treatment, mostly because they felt self-conscious or tense (psychological discomfort). OHRQoL then declined to moderately affected at the beginning of expansion with MARPE, with patients mostly having trouble with pronouncing words or a change in taste (functional limitation), psychological discomfort and physical pain.

These effects could have several reasons. The MARPE appliance covering the palate could have led to temporary sigmatism or speech difficulties and the change in taste could be due to the covering of palatal taste buds or impairment of the lingual taste buds. Furthermore, the pressure that MARPE exerted on both skeletal and dentoalveolar structures of the maxilla could be a plausible cause of physical pain, while the central diastema which appeared as a result of expansion exacerbated the existing feelings of self-consciousness. After the initial deterioration, however, OHRQoL improved during the second half of the treatment and returned to starting levels once the expansion was completed, see: Fig. 4. This graph showed weeks 1 to 5 of the expansion phase because these weeks covered the mean duration of a MARPE treatment. The sample size at weeks 6–10 was considered insufficient to draw reliable conclusions. Overall, notwithstanding the negative effects, OHRQoL was relatively high and MARPE was generally well-tolerated.

In comparison with other therapies for maxillary expansion, the findings on MARPE are largely comparable. Altieri et al. (2020) reported that there was a deterioration of OHRQoL during the expansion phase with Rapid Palatal Expansion and Alghamdi et al. (2017) found that RPE had a significant negative impact on aspects of OHRQoL, such as masticatory and speech problems [13, 20]. This worsening of the OHRQoL during RPE was of temporary nature and significantly improved once the transverse maxillary deficiency was corrected and the appliance was removed [14, 21]. Similar findings were described for surgically-assisted RPE, with the surgical procedure and ensuing expansion described as tolerable [22]. Previous research on OHRQoL and orthodontic treatment in general has reported an improvement after orthodontic treatment [9]. In the current study, however, the MARPE appliance was left in place for three months after completion of expansion and the orthodontic treatment was continued with fixed appliances, which could explain why an improvement of OHRQoL immediately after MARPE did not occur.

Furthermore, research has suggested that patient perception and satisfaction regarding the outcome of expansion, with SARPE in this specific study, were influenced by pre-treatment expectations [23]. Concordantly, clinicians should be aware of the impact of MARPE on patients’ quality of life and manage the expected discomfort and impediments with effective communication, education and setting the right expectations, which could soften the effects and improve patient perception and satisfaction.

### Pain perception

Physical pain assessed with the OHIP-14 as the frequency of painful aching and discomfort while eating was found to increase significantly during expansion with MARPE. For the evaluation of pain intensity in the context of orthodontic treatments, tooth extractions are often used as a reference procedure given the frequent occurrence and the acceptance as an intervention [24]. Ganzer et al. (2016) reported a mean VAS-score of 38.50 mm after premolar extraction while Chen et al. (2011) measured a score of 35.80 mm [24, 25]. In comparison, MARPE treatment, with a mean VAS-score of 33.72 mm for average pain during the first week of expansion, caused less pain than premolar extractions and can be considered moderately painful. Furthermore, Tseng et al. (2010) reported a pain VAS-score of 36.3 mm 24 h after placing of fixed orthodontic appliances, which is comparable to the pain MARPE caused at initiation [26]. Overall pain during MARPE, with a mean VAS-score of 16.00 mm, was mild, and after the temporary peak of pain during the first fifth of expansion, the pain decreased and remained very mild from the third fifth until the end of expansion. Regarding the intake of analgesics, they were taken by 59% of participants during the initiation of MARPE treatment, which was lower than the intake of analgesics after premolar extraction (70%) and third molar surgery (96%) but higher than after the placing of fixed orthodontic appliances (16%) [27–29].

Regarding the evolution of pain intensity during MARPE, it was similar to other non-surgical RPE treatments, which caused pain in 98% of patients, with the highest level of pain during the initial phase of expansion, mostly during the first week, after which it gradually decreased [14, 30–33]. A direct comparison with SARPE or other (bi)maxillary osteotomies was not possible, due to the fact that SARPE takes places under general anaesthesia and because random allocation of patients to a surgery and a non-surgery group may face challenging ethical issues given the invasive nature of SARPE. However, a recent literature review on post-operative pain in the sagittal split ramus osteotomy and intra oral vertical ramus osteotomy found that the mean VAS-scores of the first postoperative day were 20–53 mm in the former and 29.3–31.3 mm in the latter, which is comparable to or higher than the pain experienced during the first week of expansion with MARPE and significantly higher than the overall pain intensity of MARPE [34]. In addition, orthognathic surgery was reported to have a complication rate of 12.4% with possible complications such as impaired sensation, temporomandibular joint disorders, haemorrhage, postoperative swelling and bad split [35, 36]. Considering the advantages of MARPE, such as its non-surgical aspect and subsequent absence of surgery-related complications, it can be concluded that MARPE is a more patient friendly maxillary expansion therapy than SARPE.

### Limitations

Measurements of patient perception relied on the ability to translate a personal experience into an objective measure, which was influenced by interindividual differences, and were inherently subjective. The patients were instructed to answer the questionnaires as accurately and truthfully as possible; it was not possible to test for reliability. The absence of a control group complicated direct comparison between MARPE and RPE or SARPE. In order to facilitate a clinically relevant interpretation, the mean VAS-scores were compared to conventional orthodontic and surgical (expansion) treatments.

## Conclusions

The results of the current study suggest that MARPE is a generally well-tolerated treatment. A temporary decline in OHRQoL, in particular, functional limitation, psychological discomfort and social disability, as well as moderate pain, is present at the start of expansion. As the treatment advances, there is a recovery of OHRQoL and very mild pain, followed by a return to the initial OHRQoL when expansion is completed. Clinician awareness of these effects and adequate patient information could enhance the patient perception of MARPE.

## Data Availability

The datasets used and/or analysed during the current study are available from the corresponding author on reasonable request.

## Abbreviations

MARPE: Miniscrew-Assisted Rapid Palatal Expansion
OHRQoL: Oral Health-related Quality of Life
OHIP: Oral Health Impact Profile
OHIP-14: Short form of OHIP
OHIP-NL14: Short form of OHIP in Dutch
T0: Baseline
P1: Expansion phase
P2: Post-expansion phase
VAS: Visual Analogue Scale
RPE: Rapid Palatal Expansion
SARPE: Surgically-Assisted Rapid Palatal Expansion
IRB: Institutional Review Board
EDC: Electronic Data Capture
GDPR: General Data Protection Regulation
STROBE: STrengthening the Reporting of OBservational studies in Epidemiology
